# Destructive Photon Echo Formation in Six‐Wave Mixing Signals of a MoSe_2_ Monolayer

**DOI:** 10.1002/advs.202103813

**Published:** 2021-10-29

**Authors:** Thilo Hahn, Diana Vaclavkova, Miroslav Bartos, Karol Nogajewski, Marek Potemski, Kenji Watanabe, Takashi Taniguchi, Paweł Machnikowski, Tilmann Kuhn, Jacek Kasprzak, Daniel Wigger

**Affiliations:** ^1^ Institute of Solid State Theory University of Münster Münster 48149 Germany; ^2^ Department of Theoretical Physics Wrocław University of Science and Technology Wrocław 50‐370 Poland; ^3^ Laboratiore National des Champs Magnétiques Intenses LNCMI‐EMFL CNRS UPR3228, Univ. Grenoble Alpes, Univ. Toulouse, Univ. Toulouse 3, INSA‐T Grenoble and Toulouse France; ^4^ Central European Institute of Technology Brno University of Technology Brno Czech 61200 Republic; ^5^ Institute of Experimental Physics Faculty of Physics University of Warsaw Warszawa 02‐093 Poland; ^6^ Research Center for Functional Materials National Institute for Materials Science Tsukuba 305‐0044 Japan; ^7^ International Center for Materials Nanoarchitectonics National Institute for Materials Science Tsukuba 305‐0044 Japan; ^8^ Université Grenoble Alpes CNRS, Grenoble INP, Institut Néel Grenoble 38000 France

**Keywords:** nonlinear spectroscopy, photon echo, transition metal dichalcogenides

## Abstract

Monolayers of transition metal dichalcogenides display a strong excitonic optical response. Additionally encapsulating the monolayer with hexagonal boron nitride allows to reach the limit of a purely homogeneously broadened exciton system. On such a MoSe_2_‐based system, ultrafast six‐wave mixing spectroscopy is performed and a novel destructive photon echo effect is found. This process manifests as a characteristic depression of the nonlinear signal dynamics when scanning the delay between the applied laser pulses. By theoretically describing the process within a local field model, an excellent agreement with the experiment is reached. An effective Bloch vector representation is developed and thereby it is demonstrated that the destructive photon echo stems from a destructive interference of successive repetitions of the heterodyning experiment.

## Graphical abstract

This is the first report of a destructive photon echo effect detected in a six‐wave mixing signal from the atomically thin semiconductor MoSe_2_. By developing a sound theoretical model, it is shown that the effect is caused by the exciton‐exciton interaction in the material. It provides a new ultrafast spectroscopy tool to study the many‐particle interaction in 2D semiconductors.

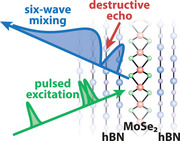

## 1 Introduction

The spin echo^[^ ^1^ ^]^ is an essential effect in nuclear magnetic resonance spectroscopy and the basis for all sorts of complex radio pulse sequences^[^ ^2^ ^]^ that are routinely applied in medicine,^[^ ^3^ ^]^ chemistry,^[^ ^4^ ^]^ or physics.^[^ ^5^, ^6^, ^7^ ^]^ While spin resonances are driven by radio frequencies, optical frequencies are required to resonantly excite typical charge transitions; in this regime the analogous phenomenon is called photon echo.^[^ ^8^ ^]^ First demonstrations were performed on ruby crystals,^[^ ^8^, ^9^ ^]^ but the photon echo spectroscopy also has a long standing history in semiconductor optics. It has been used to study different types of exciton dynamics, ranging from exciton‐exciton scattering^[^ ^10^, ^11^, ^12^, ^13^ ^]^ to exciton‐phonon coupling,^[^ ^14^, ^15^ ^]^ and it has been applied to 3D bulk,^[^ ^10^, ^16^, ^17^ ^]^ 2D quantum well,^[^ ^18^, ^19^ ^]^ 1D nanowire,^[^ ^20^ ^]^ and 0D quantum dot structures.^[^ ^21^, ^22^, ^23^, ^24^ ^]^ However, the technique is not restricted to solid state samples; it has also been applied to liquids.^[^ ^25^, ^26^, ^27^ ^]^ In its classical form, the photon echo is based on a four‐wave mixing (FWM) process and therefore it constitutes a nonlinear process of third order (χ^(3)^) in the low excitation limit.^[^ ^28^ ^]^ Especially in single low‐dimensional systems like quantum dots, it requires large effort to detect such nonlinear optical signals.^[^ ^29^, ^30^ ^]^ Interestingly, due to their strong excitonic optical response, monolayers of transition metal dichalcogenides (TMDCs) show a remarkable signal strength in FWM spectroscopy.^[^ ^31^, ^32^, ^33^, ^34^ ^]^ So far, the direct correspondence between inhomogeneous spectral broadening and photon echo duration has been used to map the inhomogeneity of TMDC monolayers.^[^ ^31^, ^32^, ^34^, ^35^ ^]^

We here exploit the strong excitonic optical response of TMDC monolayers, and explore six‐wave mixing (SWM) signals from a MoSe_2_ monolayer in the low excitation limit, which here represents the χ^(5)^‐regime.^[^ ^36^, ^37^, ^38^, ^39^ ^]^ We find that the signal dynamics exhibit a characteristic temporary suppression depending on the delay between the pulses. Supported by a theoretical model based on the local field effect describing exciton‐exciton interaction, we explain that this suppression can be understood as the formation of a destructive photon echo.

## 2 Results

### 2.1 Sample and Four‐Wave Mixing Dynamics

**Figure 1:**
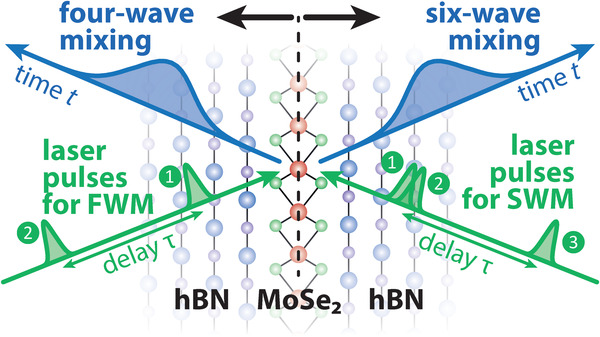
Sample structure consisting of a multilayer hBN, monolayer MoSe_2_, multilayer hBN stack. (Left) Four‐wave mixing (FWM) generated by two laser pulses with tunable delay τ. (Right) Six‐wave mixing (SWM) with three excitation pulses having a tunable delay τ between pulses 2 and 3. The FWM and SWM dynamics are measured in real time *t*.

**Figure 2:**
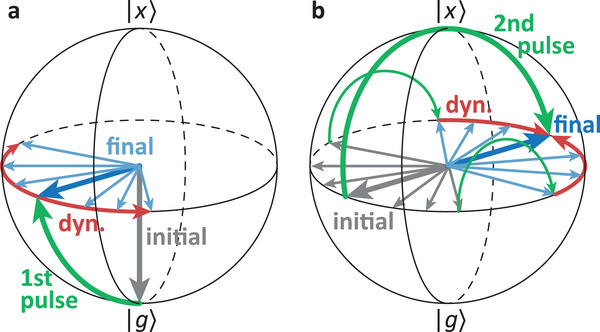
Schematic Bloch vector image of the photon echo process. a) 1st laser pulse excitation with following dissipation. b) 2nd excitation inverting all coherences and following rephasing. Laser pulse rotations are depicted in green, initial and final Bloch vectors in grey and blue, respectively, and the inhomogeneity induced coherence dynamics in red.

We perform multi‐wave mixing experiments on an hBN/MoSe_2_/hBN heterostructure as schematically depicted in **Figure** **1**. By radio frequency modulating the incoming beams, the different pulses are labeled by phases ϕ_*j*_.^[^ ^40^ ^]^ After heterodyning the emitted light with a specific *N*‐wave mixing (NWM) phase, that is a particular phase combination of the form ϕ_NWM_ = ∑_*j*_ *a*_*j*_ϕ_*j*_ ($a_{j}\in Z$, ∑_*j*_|*a*_*j*_| = *N* − 1, ∑_*j*_ *a*_*j*_ = 1), we retrieve the corresponding nonlinear signal from the investigated monolayer.^[^ ^41^ ^]^ Details are given in the experimental section. We use the same sample as investigated in ref. [35] where the echo formation in two‐pulse FWM signals with ϕ_FWM_ = 2ϕ_2_ − ϕ_1_ was used to study the inhomogeneity of the structure. The well known photon echo appears due to the dephasing impact of the structural inhomogeneity on the exciton's coherence as schematically depicted via Bloch vectors in **Figure** **2**.^[^ ^42^ ^]^ For the sake of simplicity in the illustration we show a combination of a π/2 and a π pulse, which results in a photon echo in the full coherence. However, when considering the FWM coherence characterized by the phase ϕ_FWM_ the photon echo appears for any combination of pulse areas. The first laser pulse in Figure 2a having a pulse area of θ_1_ = π/2 and arriving at the time *t* = −τ generates an exciton coherence. Because of the presence of different transition energies, originating from strain and dielectric variations, the coherences generated by the first laser pulse oscillate with different frequencies resulting in a dephasing of the total coherence. As depicted in Figure 2b, after a delay τ, that is, at the time *t* = 0, a second laser pulse with the pulse area θ_2_ = π inverts all Bloch vectors. This is followed by a rephasing of the different coherences. The rephasing takes the same time as the dephasing and therefore the FWM signal is significantly enhanced due to constructive interferences at the time precisely given by the delay time *t* = τ between the two pulses.

**Figure 3:**
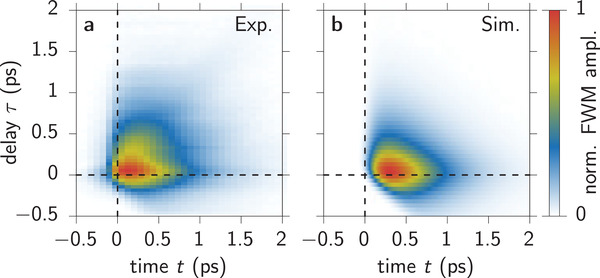
Four‐wave mixing dynamics as a function of real time *t* and delay τ. a) Experiment and b) simulation.

In **Figure** **3**a we show the measured FWM signal from our sample as a function of the real time *t* after the second pulse and the delay τ. The pulse alignment is such that the pulse with phase ϕ_1_ arrives at *t* = −τ and the pulse with ϕ_2_ at *t* = 0. We find that the amplitude is not concentrated along the diagonal, which would represent a photon echo. Instead, starting from its maximum around (τ, *t*) = (0, 0) it basically decays to positive delays and times. This demonstrates that the investigated sample position is virtually homogenous because the photon echo is not present. Figure 3b shows the corresponding simulated FWM amplitude |*S*_FWM_| within a few‐level system where we take the exciton transfer into the optically uncoupled valley as well as the exciton‐exciton interaction in terms of the local field model into account. It describes the optically induced dynamics of the exciton's coherence *p* and its occupations *n* and $n^{\prime }$ via^[^ ^11^, ^43^, ^44^ ^]^

(1a) $$\begin{array}{cc} \frac{dp}{dt} & =i\left(1-2n\right)\left[\Omega \left(t\right)+Vp\right]-\left(\beta +i\omega_{0}\right)p\; \end{array}$$

(1b) dndt=2Im[Ω∗(t)p]−Γn−λ(n−n′)

(1c) $$\begin{array}{cc} \frac{dn^{\prime }}{dt} & =-\Gamma n^{\prime }-\lambda \left(n^{\prime }-n\right)\; \end{array}$$

Here, *n* and $n^{\prime }$ are the occupations in the optically coupled and uncoupled valleys, respectively, Ω(*t*) is the time dependent Rabi frequency describing the optical excitation by co‐circlarly polarized pulses, β and Γ are the dephasing and the decay rate, respectively, ℏω_0_ is the exciton energy in the absence of the local field coupling, and *V* is the strength of the local field coupling. Compared to ref. [44] we do not find a significant impact of excitation induced dephasing as discussed in more detail in the Supporting Information. As recently studied in ref. [44] we additionally take an intervalley scattering contribution with the rate λ into account. In the special case λ = 0, the system reduces to a two‐level system. We have recently investigated the local field model in the context of nonlinear optical signals focussing on FWM^[^ ^43^ ^]^ and pump‐probe spectroscopy,^[^ ^44^ ^]^ showing that the handy description produces resonant optical spectroscopy signals that are consistent with experiments. In a nutshell, the local field effect leads to energy shifts of the exciton depending on the its occupation. In the limit of ultrafast optical pulses Equation (1) can be solved analytically relating the coherence *p* ^+^ and the occupation *n* ^+^ after the pulse to the respective values *p* ^−^ and *n* ^−^ immediately before the pulse

(2a) $$\begin{array}{cc} p^{+} & =p^{-}\cos^{2}\left(\frac{\theta }{2}\right)+\frac{i}{2}\sin\left(\theta \right)\left(1-2n^{-}\right)e^{i\phi }+\sin^{2}\left(\frac{\theta }{2}\right)p^{-*}e^{i2\phi }\\ & \approx p^{-}+i\frac{\theta }{2}\left(1-2n^{-}\right)e^{i\phi }\; \end{array}$$

(2b) n+=n−+sin2θ2(1−2n−)+sin(θ)Imp−e−iϕ≈n−+θImp−e−iϕ,

where θ is the pulse area and ϕ its phase. The approximations in Equation (2) describe the light‐field induced contributions of a single pulse in first order of the pulse area, which is sufficient for reproducing the contributions of the order *V* ^2^ to the SWM signal, discussed in the main text of this work (other contributions, involving terms up to the second order in the pulse areas, are discussed in the Supporting Information).

Starting from the excitonic ground state characterized by $n=n_{1}^{-}=0$, $p=p_{1}^{-}=0$ the first pulse in the linear order creates

(3a) $$\begin{array}{cc} p_{1}^{+} & \approx i\frac{\theta_{1}}{2}e^{i\phi_{1}}\; \end{array}$$

(3b) $$\begin{array}{cc} n_{1}^{+} & \approx 0\; \end{array}$$

which shows that relevant exciton occupations will only be created by a second laser pulse from $p_{1}^{+}$. Our goal is to derive the nonlinear optical response in the lowest order of the pulse area θ because the experiments are performed with low pulse powers. In the case of the SWM signal this is the fifth order $O(\theta^{5})$ (χ^(5)^‐regime).

Once optical pulses have generated coherences *p*_0_ and occupations *n*_0_ the corresponding free propagation [Ω = 0, in Equation (1)] is governed by pure dephasing of *p*, exciton decay and intervalley scattering of $n^{\left(\prime \right)}$, and local field coupling between *p* and *n*. The corresponding dynamics can also be calculated analytically. Focusing on the optically addressed occupation first, its time‐dependence reads

(4a) $$\begin{array}{cc} n(t) & =\frac{n_{0}+n_{0}^{\prime }}{2}e^{-\Gamma t}+\frac{n_{0}-n_{0}^{\prime }}{2}e^{-(\Gamma +2\lambda )t}\; \end{array}$$

which leads to a balanced occupation between the two valleys *n* and $n^{\prime }$ on the timescale 1/(2λ) and a decay of both occupations with the rate Γ. As known from literature^[^ ^44^, ^45^, ^46^ ^]^ and as considered in this work the intervalley scattering is typically significantly faster than the decay, that is, Γ ≪ 2λ. As a simplifying approximation for the sake of interpreting the results we can therefore assume that the occupations are balanced rapidly after an optical pulse, that is,

(4b) $$\begin{array}{cc} n(t) & \approx \frac{n_{0}+n_{0}^{\prime }}{2}e^{-\Gamma t}\; \end{array}$$

This step might lead to slight deviations for short delays in the range τ ≈ 1/(2λ) which are however hardly visible for the parameters chosen here as shown in the Supporting Information. Further we can only optically manipulate the occupation *n*, while $n^{\prime }$ remains unchanged by the applied laser pulses. According to Equation (3a) these pulses moreover add phase labels ϕ. As explained at the beginning of this section and as practically applied below, we are only interested in specific phase combinations that describe the considered wave‐mixing signal. Consequently, any change of the occupation *n* is labeled by phase factors which do not apply to the other valley $n^{\prime }$. Therefore, the latter is irrelevant for the final optical signal and we can consider $n_{0}^{\prime }=0$ in Equation (4b) resulting in

(4c) $$\begin{array}{cc} n(t) & \approx \frac{n_{0}}{2}e^{-\Gamma t}\; \end{array}$$

We want to remark that in the opposite limit of a very slow scattering rate 2λ ≪ Γ the occupation dynamics would directly be given by *n*(*t*) = *n*_0_ *e* ^−Γ*t*^. In this case all later discussions would work in the same way. By replacing *V* → 2*V* in all the following derivations, one can even directly retrieve the corresponding equations.

Based on this approximation for the occupation dynamics we can calculate the coherence dynamics in the frame rotating with *ω*_0_−*V*

(5) $$\begin{array}{ccc} p(t) & = & p_{0}\exp\left[-i\frac{2V}{\Gamma }\underset{0}{\int^{t}}dt^{\prime }\;n\left(t^{\prime }\right)\right]e^{-i\beta t}\\ & \approx & p_{0}\exp\left[-i\frac{2V}{\Gamma }\frac{n_{0}}{2}\left(1-e^{-\Gamma t}\right)\right]e^{-i\beta t}\\ & \approx & p_{0}e^{iVn_{0}t}e^{-i\beta t}\\ & \approx & p_{0}\left[1-iVn_{0}t-\frac{1}{2}{\left(Vn_{0}t\right)}^{2}\right]e^{-i\beta t} \end{array}$$

In the first approximation step, we have used the approximated occuption from Equation (4c). As the exciton decay is much slower than the dephasing Γ ≪ β, in the second step we take Γ → 0. Finally, we keep the local field‐induced contributions up to the second order in the exciton occupation, that is, considering *Vn*_0_ *t* ≪ 1. Below we will see that these are the terms which contribute to the SWM signal in the χ^(5)^‐regime. In the Supporting Information, we show that only terms up to $O(V^{2})$ contribute to the χ^(5)^‐regime of the SWM signal, while contributions with higher powers in *V* appear in higher orders of the optical field. The last equation tells us that the local field induced mixing of the occupation *n*_0_ with the coherence *p*_0_ into the coherence *p*(*t*) in the lowest order grows linearly in time with a rate given by *n*_0_ and the local field strength *V*. We will use this argument later on.

We simulate the FWM signal with the phase combination 2ϕ_2_ − ϕ_1_ by calculating the coherence dynamics *p*(*t*) following a two‐pulse sequence and filter this quantity with respect to the required phase combination as described in refs. [43, 44]. With this we find the signal dynamics $|S_{\mathrm{FWM}}{\left|\approx \right|}^{2\phi_{2}-\phi_{1}}p_{2}\left(t,\tau \right)|$ depicted in Figure 3b. To achieve the excellent agreement with the experiment in Figure 3a, we used a local field strength of *V* = 100 ps^−1^ and pulse areas of θ_1_ = θ_2_/2 = θ = 0.02π, a Gaussian pulse duration of Δ*t* = 70 fs (standard deviation), a dephasing rate of β = 3 ps^−1^, an intervalley scattering rate λ = 4 ps^−1^, and a decay rate of Γ = 0.6 ps^−1^. Note that the depicted signal was calculated numerically because we considered a non‐vanishing pulse duration and we did not employ the approximations mentioned in Equations (2)–(5). To set the strength of the local field coupling into context, typical values for GaAs quantum wells were reported in the range of a few meV,^[^ ^47^, ^48^ ^]^ which is at least one order of magnitude smaller than considered here.

### 2.2 Six‐Wave Mixing Dynamics

**Figure 4:**
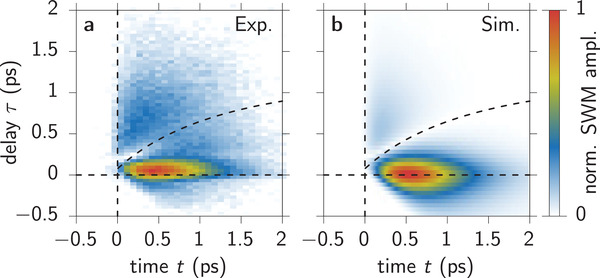
Six‐wave mixing dynamics as a function of real time *t* and delay τ. a) Experiment, b) simulation, with the curved dashed line depicting Equation (9).

In the present study we go one step further in multi‐wave mixing and consider one of the possible SWM signals generated by three laser pulses, namely ϕ_SWM_ = 2ϕ_3_ − 2ϕ_2_ + ϕ_1_. In principle there are two delays in this pulse sequence that could be varied but, as schematically shown in Figure 1 (right), we set the delay between the first two pulses to τ_12_ = 0 and only vary the second one τ_23_ = τ. The impact of a non‐vanishing τ_12_ is discussed in the Supporting Information. In the pure two‐level system this configuration probes the polarization, that is, it contains the same information as the FWM signal discussed before as shown in the Supporting Information. The measured SWM dynamics as a function of the real time *t* after the third pulse and the delay τ are shown in **Figure** **4**a. Here, the two pulses with ϕ_1_ and ϕ_2_ arrive at *t* = −τ and the pulse with ϕ_3_ at *t* = 0. The signal consists of a strong maximum at small *t* ≈ 0.5 ps and τ ≈ 0. Moving to negative delays τ < 0 (pulse 3 is arriving before 1 and 2), the signal is strongly damped. For positive delays τ > 0 it decays much slower on the same timescale as the FWM signal in Figure 3. We find a remarkable depression of the signal that stretches along the curved diagonal given by Equation (9) (dashed black line), as will be derived below. In correspondence with the previously described constructive signal enhancement in the photon echo, we call this pronounced signal reduction a destructive photon echo. Later, we will discuss criteria justifying the labeling of this feature as an echo effect.

To identify the origin of this peculiar dynamical feature we model the SWM signal within the local field model described above. We consider the same system parameters as for the FWM signal but choose equal pulse areas for all three pulses θ_1_ = θ_2_ = θ_3_ = θ = 0.02π, in agreement with the experiment. In the Supporting Information, it is shown that the exact choice of θ in the low excitation regime does not change the SWM signal dynamics. The SWM signal is extracted via the phase combination 2ϕ_3_ − 2ϕ_2_ + ϕ_1_ and the signal dynamics $|S_{\mathrm{SWM}}{\left|\approx \right|}^{2\phi_{3}-2\phi_{2}+\phi_{1}}p_{3}\left(t,\tau \right)|$ are depicted in Figure 4b. The retrieved signal agrees very well with the respective experiment in Figure 4a showing the same characteristic suppression of the signal, that is, the destructive photon echo. We give an overview regarding the impact of the different parameters on the SWM signal in the Supporting Information. Most importantly, it is shown in the Supporting Information, that the SWM dynamics do not exhibit the suppression for small local field strengths *V* demonstrating that this feature is a result of exciton‐exciton interaction. Other specific features in the signal's dynamics like the asymmetric decay between positive and negative delays or between τ and *t* were studied in detail in ref. [43] and behave similarly in SWM. The destructive photon echo effect should also be present in an inhomogeneously broadened system, where also a traditional constructive photon echo develops. As the constructive echo selects only a specific time interval of the emitted signal around *t* = τ, it leads to a suppression of the entire SWM signal for all other times. Consequently, the SWM signal and therefore the destructive photon echo, which bends away from the diagonal (discussed later), are only visible in the vicinity of *t* = τ. This aspect is studied in more detail in the Supporting Information.

**Figure 5:**
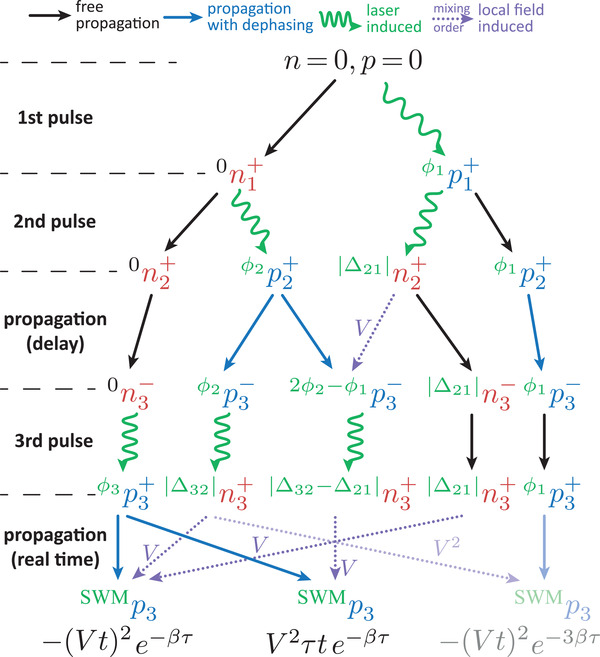
Flow chart for the three main contributions to the SWM signal listing intermediate phase‐filtered coherences (*p*, blue) and occupations (*n*, red). Green waved arrows show pulse induced, violet dotted ones local field induced, black ones free dynamics without, and blue ones with dephasing. The phase differences are defined as Δ_*nm*_ = ϕ_*n*_ − ϕ_*m*_. The flow chart holds for any order of the optical field. However, in the linear response regime considered here, it is ${}^{0}n_{1}^{+}={}^{0}n_{2}^{+}={}^{0}n_{3}^{-}=0$.

Note, that the simulation shown in Figure 4b takes the non‐vanishing pulse duration into account and is therefore performed numerically. In the limit of ultrafast laser pulses we can find analytical expressions for the SWM signal. Given that the experiment is carried out with weak pulse powers, we restrict the following studies on the lowest order in the optical field which is the χ^(5)^‐regime. In this order we have already eight different contributions as derived in the Supporting Information. From these we will focus on the ones with the strongest local field contribution which is $O(V^{2})$, that is, we omit all terms $O(V^{1})$ and $O(V^{0})$. The reason for this is the absence of the destructive echo for small *V*. In ref. [43] we have derived a flow chart representation for the construction of nonlinear wave mixing signals. In **Figure** **5** we employ this procedure to disentangle the origin of the different contributions to the SWM signal. The flow chart only shows coherences *p* (blue) and occupations *n* (red) with phase combinations (green, given as left indices) relevant for the final signal; corresponding flow charts for the contributions with $O(V^{1})$ and $O(V^{0})$ are provided in the Supporting Information. Conveniently, the $O(V^{2})$‐contributions can be derived with the approximations given in Equation (2). Note, that we introduce phase differences Δ_*nm*_ = ϕ_*n*_ − ϕ_*m*_ here. The right lower index refers to the pulse number, while the upper − (+) indicates times immediately before (after) that pulse. Starting from the excitonic ground state with *n* = 0, *p* = 0 the first pulse generates the occupation ${}^{0}n_{1}^{+}$ and the coherence ${}^{\phi_{1}}p_{1}^{+}$. Note, that in the scheme we do not restrict ourselves to the lowest order contributions in the light field and thus include also the occupation which is of second order in the pulse amplitude. The second pulse arrives at the same time (τ_12_ = 0) and creates two occupations ${}^{0}n_{2}^{+}$ and ${}^{|\Delta_{21}|}n_{2}^{+}$ and the coherence ${}^{\phi_{2}}p_{2}^{+}$. During the following propagation for the time of the delay τ two relevant things happen: On the one hand all coherences experience dephasing (blue arrows). On the other hand ${}^{\phi_{2}}p_{2}^{+}$ is mixed with ${}^{|\Delta_{21}|}n_{2}^{+}$ via the local field coupling resulting in the coherence ${}^{2\phi_{2}-\phi_{1}}p_{3}^{-}$ before the third pulse. Note, that this contribution carries the FWM phase ϕ_FWM_ = 2ϕ_2_ − ϕ_1_ which we will come back to below. After the final third pulse we have five relevant terms^[^ ^49^ ^]^: The coherence ${}^{\phi_{3}}p_{3}^{+}$ and the three occupations ${}^{|\Delta_{32}|}n_{3}^{+}$, ${}^{|\Delta_{32}-\Delta_{21}|}n_{3}^{+}$, and ${}^{|\Delta_{21}|}n_{3}^{+}$. The polarization ${}^{\phi_{1}}p_{1}^{+}$ is not affected by the second and third pulse and just evolves into ${}^{\phi_{1}}p_{3}^{+}$. During the remaining propagation step in real time *t* the three relevant SWM contributions are generated by local field mixing processes according to Equation (5).

Considering the contribution on the right first, we have to mix ${}^{\phi_{1}}p_{3}^{+}$ with ${}^{|\Delta_{32}|}n_{3}^{+}$ twice. This results in the phase combination ϕ_1_ + 2(ϕ_3_ − ϕ_2_) which is the SWM phase combination. According to Equation (5), each of these local field mixing processes contributes with an amplitude of *Vt* resulting in the amplitude (*Vt*)^2^. In addition the amplitude is damped due to the dephasing happening during the delay. Following the two paths in the diagram back to this propagation step we find that ${}^{\phi_{1}}p_{2}^{+}\;\to {\;}^{\phi_{1}}p_{3}^{-}$ and ${}^{\phi_{2}}p_{2}^{+}\;\to {\;}^{\phi_{2}}p_{3}^{-}$ contribute with a dephasing term ∼*e* ^−βτ^. The latter one is used twice due to the double local field mixing resulting in the total damping rate of *e* ^−3βτ^.

Moving on to the left contribution in Figure 5, we find that in the last propagation the coherence ${}^{\phi_{3}}p_{3}^{+}$ is local‐field mixed with ${}^{|\Delta_{32}|}n_{3}^{+}$ and ${}^{|\Delta_{21}|}n_{3}^{+}$ once, resulting in the SWM phase combination ϕ_3_ + (ϕ_3_ − ϕ_2_) − (ϕ_2_ − ϕ_1_). Each of these processes contributes an amplitude of *Vt* again resulting in (*Vt*)^2^. The difference to the first term is that here only ${}^{|\Delta_{32}|}n_{3}^{+}$ stems from a coherence, namely ${}^{\phi_{2}}p_{2}^{+}$ which experiences a dephasing during the delay. Consequently, the entire contribution is damped by *e* ^−βτ^. This already shows that this contribution is more important for the final SWM signal than the right term discussed first.

The final main contribution is the middle one in Figure 5. Here, in the last real time propagation the local field mixing happens between ${}^{\phi_{3}}p_{3}^{+}$ and ${}^{|\Delta_{32}-\Delta_{21}|}n_{3}^{+}$ which contributes with an amplitude of *Vt*. Following the path back, we find that the occupation is created from the polarization ${}^{2\phi_{2}-\phi_{1}}p_{3}^{-}$ which itself is produced by a local field mixing step between ${}^{\phi_{2}}p_{2}^{+}$ and ${}^{|\Delta_{21}|}n_{2}^{+}$. The mixing process lasts for the delay time and therefore contributes with a factor *V*τ to the final SWM amplitude. This is also the step where the only dephasing happens resulting in a final amplitude of *V* ^2^τ*te* ^−βτ^.

**Figure 6:**
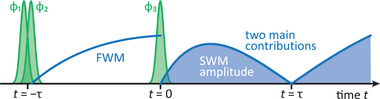
Schematic picture of the pulse sequence and signal development from the two main contributions.

Directly comparing the three contributions in Figure 5 we find that the right one is smaller than the other two due to the stronger dephasing during the delay. We will therefore disregard this term from now on. The other two terms are of the order *V* ^2^ and exhibit the same dephasing with *e* ^−βτ^. From the full derivation given in the Supporting Information we find that these two terms in Figure 5 carry opposite signs. We will explain these signs later when introducing an effective Bloch vector picture to illustrate the destructive echo formation. Finally, adding the two terms we get

(6) $$\begin{array}{c} {}^{\mathrm{SWM}}p_{3}\left(t,\tau \right)∼V^{2}\left(\tau t-t^{2}\right)e^{-\beta (\tau +t)} \end{array}$$

where we also added the dephasing rate for the propagation in *t* after the last pulse. We find that the two terms exactly compensate each other for *t* = τ. This renders our first step toward the understanding of the destructive echo. We identified two paths (signal contributions) with the same damping but with different magnitudes depending on the delay τ that act destructively in the total SWM signal. To illustrate the interplay between these two terms in **Figure** **6** we schematically plot the applied laser pulses and the absolute value of the final SWM signal retrieved from the previous derivations. We also include the intermediate growth of the FWM coherence lasting for the delay τ.

The full SWM signal in the χ^(5)^‐regime is derived in the Supporting Information and reads

(7) $$\begin{array}{c} \begin{array}{c} {}^{\mathrm{SWM}}p_{3}\left(t,\tau \right)={\left(\frac{\theta }{2}\right)}^{5}\left[i+V\left(\tau -4t\right)-\frac{i}{2}{\left(Vt\right)}^{2}e^{-2\beta \tau }+iV^{2}t\left(\tau -t\right)\right]e^{-\beta (t+\tau )} \end{array} \end{array}$$

Interestingly, we also find a suppression of the signal when only considering the terms $O(V^{1})$, namely for *t* = τ/4. We obviously do not find such a feature in our measurement, which shows that the linear order in *V* does not have a significant contribution.

**Figure 7:**
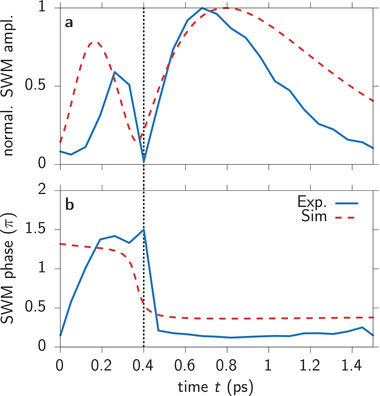
Amplitude and phase dynamics of the destructive echo. a) SWM amplitude dynamics for a delay of τ = 0.35 ps. Experiment as solid blue and simulation as dashed red line. b) Respective dynamics of the phase of the SWM signal.

One advantage of the spectral interferometry in the applied approach is the possibility to detect—besides the amplitude—also the phase of the SWM signal.^[^ ^50^ ^]^ We see that for *t* < τ the positive contribution ∼τ*t* dominates while for *t* > τ the negative one ∼−*t* ^2^ is larger. Therefore we expect a phase jump when crossing the destructive echo in time *t* < τ → *t* > τ. To confirm this in **Figure** **7**a, we plot the measured (solid blue) and calculated (dashed red) SWM signal amplitude as a function of time *t* for τ = 0.35 ps. We slightly adjusted the pulse duration to Δ*t* = 80 fs to achieve the good agreement with this experiment. The finding that the simulated signal does not drop to zero shows the impact of the non‐compensating contributions, like the ordinary SWM signal in Equation (7), and the influence of the considered non‐vanishing pulse duration. In Figure 7b we show the corresponding SWM phase from *S*_SWM_ = |*S*_SWM_|*e* ^*i*φ^. We indeed find a jump of approximately π at the destructive echo as would be expected for the two dominant terms. However, all other contributions, which are naturally present in the numerical simulation, lead to a reduction of the phase jump and a further distortion of the destructive echo dynamics. We again find that the full depression of the signal happens at *t* ≈ 0.4 ps which is slightly later than *t* = τ = 0.35 ps. This slight delay of the effect mainly stems from the non‐vanishing decay rate of the exciton Γ ≠ 0.

To include the exciton decay we have to go back to the full dynamics in Equation (5) and directly consider *Vn*_0_(1 − *e* ^−Γ*t*^)/Γ ≪ 1 without taking the limit Γ → 0 leading to

(8a) $$\begin{array}{cc} n(t) & =n_{0}e^{-\Gamma t}\; \end{array}$$

(8b) $$\begin{array}{cc} p(t) & \approx p_{0}\left[1-i\frac{V}{\Gamma }n_{0}\left(1-e^{-\Gamma t}\right)-\frac{V^{2}}{2\Gamma^{2}}n_{0}^{2}{\left(1-e^{-\Gamma t}\right)}^{2}\right] \end{array}$$

Now we consider the different paths in Figure 5 to determine the decay's impact on the different SWM contributions. The term ∼*t* ^2^ stems from the delay step ${}^{|\Delta_{21}|}n_{2}^{+}\;\to {\;}^{|\Delta_{21}|}n_{3}^{-}$, contributing a factor *e* ^−Γτ^, and the final double wave mixing step gives (1 − *e* ^−Γ*t*^)^2^. The τ*t‐*contribution has a local‐field mixing step during the delay leading to (1 − *e* ^−Γτ^) and only a single local field mixing after the last pulse, that is, (1 − *e* ^−Γ*t*^). Finally, the two contributions compensate each other for

(9) $$\begin{array}{c} {\left(1-e^{-\Gamma t}\right)}^{2}e^{-\Gamma \tau }=\left(1-e^{-\Gamma t}\right)\left(1-e^{-\Gamma \tau }\right)\;\\ \Rightarrow \tau =\frac{1}{\Gamma }\ln\left(2-e^{-\Gamma t}\right)\; \end{array}$$

We find that the decay additionally acts on the local field mixing processes because it dynamically reduces the occupation during the mixing. This affects the *t* ^2^‐contribution in a different way than the part ∼τ*t* and the destructive echo gets delayed with respect to *t* = τ. Equation (9) does not depend on *V* as confirmed in the Supporting Information. Consequently, it also holds in both limiting cases of small and large intervalley scattering as discussed below Equation (4c) and can be expected to be correct also for intermediate λ‐values. Note, that the curve describing the dynamics of the SWM signal depression in Figure 4 follows Equation (9) and is additionally shifted by the pulse width Δ*t* to shorter times to compensate the non‐vanishing pulse duration. The curve almost perfectly follows the distribution of the destructive echo.

### 2.3 Quasi‐Bloch Vector and Phase Inhomogeneity

As discussed in the previous section, the FWM‐polarization constitutes the basis for the SWM signal. Therefore, it is instructive to introduce the concept of the quasi‐Bloch vector to explain the wave‐mixing origin for the FWM signal in the next section. Building on this picture we will be able to explain the destructive photon echo formation in the SWM signal in the following section.

#### 2.3.1 Four‐Wave Mixing Quasi‐Bloch Vector

The ordinary echo in FWM is most instructively visualized within the Bloch vector picture in Figure 2 where the inhomogeneity leads to different rotation speeds of the various Bloch vector realizations. In that case each exciton energy is represented by a single Bloch vector. Here, we are dealing with a slightly different situation. The wave mixing experiment is repeated numerous times with successively different phase combinations (ϕ_1_, ϕ_2_, ϕ_3_) for each repetition of the measurement. Therefore we have to represent each run of the experiment, that is, each phase combination, by a single Bloch vector. To find the realized Bloch vectors we have a look at the coherence and occupation immediately after the second pulse^[^ ^43^ ^]^

(10a) $$\begin{array}{cc} p_{2}^{+} & =i\frac{\theta }{2}\left[e^{i\phi_{1}}+e^{i\phi_{2}}-\frac{\theta^{2}}{4}e^{i(2\phi_{2}-\phi_{1})}\right]+O\left(\theta^{5}\right) \end{array}$$

(10b) $$\begin{array}{cc} n_{2}^{+} & =\frac{\theta^{2}}{2}\left[1+\cos\left(\phi_{2}-\phi_{1}\right)\right]+O\left(\theta^{4}\right)\; \end{array}$$

where we consider all terms up to the third order in the optical field (χ^(3)^‐regime) because our measurements are performed with low excitation powers. Following the flow chart in Figure 5 we find that the two terms ${}^{\phi_{2}}p_{2}^{+}$ and ${}^{|\Delta_{21}|}n_{2}^{+}$ perform local field induced mixing during the following propagation before the third pulse (delay step). Exactly this term was identified as a contribution of the FWM signal in ref. [43]. Therefore, we will study this process in more detail. Our goal is to isolate a set of Bloch vectors that allows us to directly extract the final wave mixing signal by integrating over the entire set. To achieve this we have to already filter the coherence with the respective wave mixing phase factor. In this case we are interested in the FWM contribution, which means that we have to consider the phase factor $e^{i\phi_{\mathrm{FWM}}}=e^{i(2\phi_{2}-\phi_{1})}$ and consequently the filtered polarization ${}^{\phi_{2}}p_{2}^{+}\;e^{-i\phi_{\mathrm{FWM}}}$. This brings us to the two relevant terms

(11a) $$\begin{array}{cc} & \tilde{p}={}^{\phi_{2}}p_{2}^{+}\;e^{-i\phi_{\mathrm{FWM}}}=i\frac{\theta }{2}e^{-i(\phi_{2}-\phi_{1})}\; \end{array}$$

(11b) $$\begin{array}{cc} & \tilde{n}={}^{|\Delta_{21}|}n_{2}^{+}=\frac{\theta^{2}}{2}\cos\left(\phi_{2}-\phi_{1}\right)\; \end{array}$$

We use these expressions to define a *quasi‐Bloch vector* for this FWM contribution via

**Figure 8:**
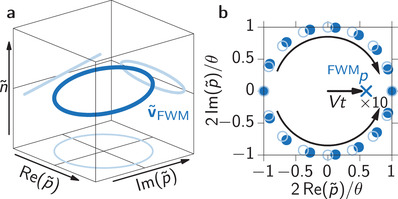
Quasi‐Bloch vector of the FWM contribution. a) Lissajous curve of the initial distribution of quasi‐Bloch vectors. b) Dynamics of the quasi‐Bloch vectors. Initial distribution as bright open circles and for a time *t* > 0 after the second pulse as filled dark circles. The final integrated FWM coherence ^FWM^ *p* is marked as blue cross and its propagation is given by *Vt*.

(12) v∼FWM(t=0)=Re(p∼)Im(p∼)n∼=θ2sin(Δ21)sin(Δ21+π/2)θsin(Δ21+π/2)

with the previously introduced phase difference Δ_21_ = ϕ_2_ − ϕ_1_. We call this quantity quasi‐Bloch vector because it does not represent the entire density matrix, as the full Bloch vector does. When varying Δ_21_ as it is done in experiment and simulation, we already see that this set of quasi‐Bloch vectors follows a 3D Lissajous curve with a frequency relationship of 1:1:1 as depicted in **Figure** **8**a. It appears as a circle tilted around the Re($\tilde{p}$)‐axis and its projections on the different planes of the coordinate system form Lissajous curves with 1:1 frequency relations. This results in a distribution of occupations $\tilde{n}$, whose spread, represented by the tilt angle, is given by the considered pulse areas.

Starting from this distribution of quasi‐Bloch vectors directly after the second laser pulse their dynamics is governed by the local field mixing between $\tilde{p}$ and $\tilde{n}$ described by [see Equation (5)]

(13) $$\frac{d\tilde{v}}{dt}=\tilde{v}\times \left(\begin{array}{c} 0\\ 0\\ V\tilde{n} \end{array}\right)\;$$

These dynamics are a rotation of the coherence $\tilde{p}$ depending on the occupation $\tilde{n}$. Particularly important is that the rotation flips its direction for opposite signs of $\tilde{n}$. At the same time $\tilde{n}$ remains unaffected. Finally, from the distribution of quasi‐Bloch vectors at a given time $\tilde{v}\left(t\right)$ we can directly retrieve the final wave mixing contribution by integrating over all possible phase combinations, that is, over Δ_21_. We directly see that the integral over $\tilde{n}$ always vanishes and we are left with the final FWM coherence

(14) $${}^{\mathrm{FWM}}p\left(t\right)=\int_{0}^{2\pi }d\Delta_{21}\;\tilde{p}\left(t;\Delta_{21}\right)\;$$

For the initial distribution of quasi‐Bloch vectors in Equation (12) we find that the final FWM coherence vanishes because all vectors are equally distributed on the tilted circle in Figure 8a. However, the following dynamics lead to a non‐vanishing FWM contribution. The rotation of $\tilde{p}$ with frequencies proportional to $\tilde{n}$ has two crucial consequences: i) The presence of a distribution of $\tilde{n}$ results in different frequencies. As already discussed in ref. [43] this means that each run of the experiment results in a slightly different emission energy of the exciton and consequently leads to a broadened FWM spectrum. Because the distribution of $\tilde{n}$ stems from the variation of the applied laser pulse phase combinations we call this spectral broadening local field induced phase inhomogeneity. ii) According to Equation (13) the sign of $\tilde{n}$ determines the rotation direction of the quasi‐Bloch vectors. The initial distribution of quasi‐Bloch vectors in Figure 8a is a circle that is tilted around the Re$\left(\tilde{p}\right)$‐axis and all points on this axis are not affected by the local field induced rotation ($\tilde{n}=0$). This axis remains a symmetry axis also for the dynamics of the quasi‐Bloch vectors: one half of the circle rotates clockwise, the other counter‐clockwise in exactly the same way. Consequently, when integrating over the relative phase Δ_21_ the opposing Im$\left(\tilde{p}\right)$ contributions compensate each other and the final FWM coherence is real, that is, Re(p∼)≠0. Because only Re($\tilde{p}$) will later contribute to the FWM signal, the crucial quantity determining the propagation direction of Re($\tilde{p}$) is n∼Im(p∼) as can be seen in Equation (13). In Figure 8b we show the quasi‐Bloch vector dynamics in the Re($\tilde{p}$),Im($\tilde{p}$)‐plane after the second pulse. The bright open circles represent the initial homogeneous distribution and the filled dark circles the distribution for a time *t* > 0. Looking at the initial distribution in Figure 8a we find that the semicircle with Im($\tilde{p})<0$ also has $\tilde{n}<0$ and the other semicircle with Im($\tilde{p})>0$ has $\tilde{n}>0$. This means that the former rotate clockwise, while the latter rotate counter‐clockwise as indicated by the curved black arrows in Figure 8b. Consequently, the weight of all quasi‐Bloch vectors, which is depicted by the blue cross, moves into the positive Re($\tilde{p}$)‐direction. The speed of this movement is given by *V* and the tilt angle of the initial quasi‐Bloch vector distribution. This illustrates that the FWM contribution grows in time and that the local field induced phase inhomogeneity governs this process.

#### 2.3.2 Six‐Wave Mixing Quasi‐Bloch Vector

Equipped with the quasi‐Bloch vector picture we can retrieve the two most important SWM signal contributions by carrying out an analogue discussion. According to Figure 5, from all possible contributions after the third laser pulse, we only need the coherence ${}^{\phi_{3}}p_{3}^{+}$ and the occupations ${}^{|\Delta_{32}|}n_{3}^{+}$, ${}^{|\Delta_{32}-\Delta_{21}|}n_{3}^{+}$, and ${}^{|\Delta_{21}|}n_{3}^{+}$. Omitting all other terms we get

(15a) $$\begin{array}{cc} p & \to i\frac{\theta }{2}e^{i\phi_{3}}\; \end{array}$$

(15b) $$\begin{array}{cc} n & \to \left[\frac{\theta^{2}}{2}\cos\left(\Delta_{32}\right)+\frac{\theta^{2}}{2}\cos\left(\Delta_{21}\right)-\frac{\theta^{4}}{8}V\tau \sin\left(\Delta_{32}-\Delta_{21}\right)\right]\; \end{array}$$

To generate the SWM contribution ∼τ*t* we need to local‐field mix the coherence filtered with respect to the SWM phase combination 2ϕ_3_ − 2ϕ_2_ + ϕ_1_ with the last term of the occupation in Equation (15b), that is,

(16a) $$\begin{array}{cc} {}^{\phi_{3}}p_{3}^{+}e^{-i\phi_{\mathrm{SWM}}} & =i\frac{\theta }{2}e^{-i(\Delta_{32}-\Delta_{21})}\; \end{array}$$

(16b) $$\begin{array}{cc} {}^{|\Delta_{32}-\Delta_{21}|}n_{3}^{+} & =-\frac{\theta^{4}}{8}V\tau \sin\left(\Delta_{32}-\Delta_{21}\right)\; \end{array}$$

From this we can directly read the respective initial quasi‐Bloch vector

(17) $${\tilde{v}}_{\mathrm{SWM},\tau t}\left(t=0\right)=\frac{\theta }{2}\left(\begin{array}{c} \sin(\alpha )\\ \sin(\alpha +\pi /2)\\ -\frac{1}{4}\theta^{3}V\tau \sin\left(\alpha \right) \end{array}\right)\;$$

with α = Δ_32_ − Δ_21_. We see that this is the same Lissajous curve structure as in the previously discussed FWM case. The only differences are that the phase variation is now given by Δ_32_ − Δ_21_, which still uniformly covers all angles, a tilt around the Im($\tilde{p}$)‐axis, and a tilt angle such that the occupation distribution $\tilde{n}$ has an amplitude of −θ^4^ *V*τ/8. The subsequent free dynamics are again governed by Equation (13).

Before discussing the shape and dynamics of this quasi‐Bloch vector set we consider the other relevant SWM contribution ∼*t* ^2^. Therefore, we have a look at

(18a) $$\begin{array}{cc} {}^{\phi_{3}}p_{3}^{+}e^{-i\phi_{\mathrm{SWM}}} & =i\frac{\theta }{2}e^{-i(\Delta_{32}-\Delta_{21})}\; \end{array}$$

(18b) $$\begin{array}{cc} {}^{|\Delta_{32}|}n_{3}^{+}{+}^{|\Delta_{21}|}\;n_{3}^{+} & =\theta^{2}\cos\left(\frac{\Delta_{32}-\Delta_{21}}{2}\right)\cos\left(\frac{\Delta_{32}+\Delta_{21}}{2}\right)\; \end{array}$$

We are finally only interested in the coherence ^SWM^ *p*_3_ describing the SWM signal. So, at this point we can already perform the integration

(19) $$\begin{array}{cc} & \int_{-\pi }^{\pi }d\left(\Delta_{32}+\Delta_{21}\right)\theta^{2}\cos\left(\frac{\Delta_{32}-\Delta_{21}}{2}\right)\cos\left(\frac{\Delta_{32}+\Delta_{21}}{2}\right)\\ & =4\theta^{2}\cos\left(\frac{\Delta_{32}-\Delta_{21}}{2}\right)\; \end{array}$$

This leaves us with the quasi‐Bloch vector for this SWM contribution reading

(20) $${\tilde{v}}_{\mathrm{SWM},t^{2}}\left(t=0\right)=\frac{\theta }{2}\left(\begin{array}{c} \sin(\alpha )\\ \sin(\alpha +\pi /2)\\ 8\theta \sin\left(\alpha /2+\pi /2\right) \end{array}\right)\;$$

with α = Δ_32_ − Δ_21_, which is obviously a 3D Lissajous curve with the frequency ratio 2:2:1.

**Figure 9:**
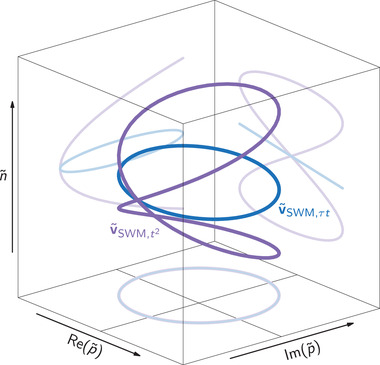
Quasi‐Bloch vector distributions after the 3rd laser pulse. The *t* ^2^‐contribution (violet) and τ*t* (blue) form Lissajous curves with frequency ratios 2:2:1 and 1:1:1, respectively.

**Figure 10:**
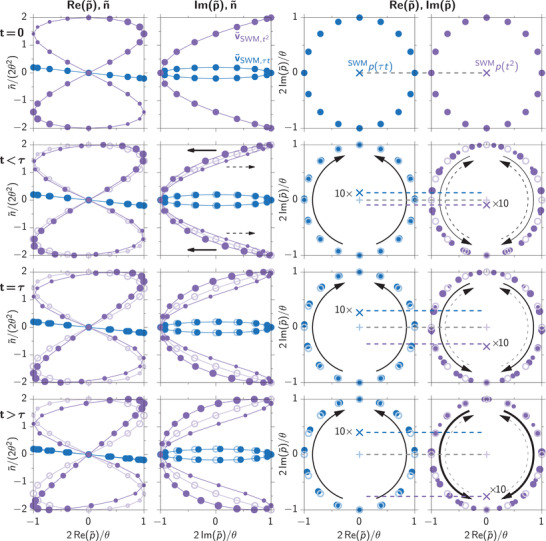
Projections of the quasi‐Bloch vector representation of the destructive echo formation. Columns show projections of the quasi‐Bloch vector defined in Equation (14) as listed on the top, the rows show different real times as given on the left. The contribution ∼*t*τ is shown as blue, ∼*t* ^2^ as violet dots. The size of the violet dots represents the product n∼Re(p∼), positive values are larger and negative ones smaller. The situation for *t* = 0 is shows as bright circles for all other times. The black arrows indicate the movement of the quasi‐Bloch vectors, solid (dashed) for positive (negative) n∼Re(p∼). The final SWM coherences are marked as crosses in the two right columns.

The two initial distributions of quasi‐Bloch vectors given in Equations (17) and (20) are depicted in **Figure** **9** in blue and violet, respectively. We vividly see the Lissajous curves leading to the distributions in $\tilde{n}$‐direction. While the 3D perspective of the curves is appealing, we will consider the projections to the three different planes of the coordinate system when discussing the dynamics of the quasi‐Bloch vectors, which are depicted in pale colors. These projections are shown in **Figure** **10**, where different times are sorted in rows (increasing from top to bottom) and different perspectives in columns.

First considering the τ*t‐*contribution (blue) we find essentially the same situation as for the intermediate FWM contribution discussed in Figure 8. The only important difference is that for the SWM contribution the circle is tilted around the Im($\tilde{p}$)‐axis. Following the same arguments as before the quasi‐Bloch vector realizations move into the positive Im($\tilde{p}$)‐direction. This movement is shown in the second column from the right in Figure 10. The initial homogeneous distribution is depicted by bright empty circles and for the considered times (written on the left) as filled dark circles. The movement is marked by the curved black arrow and the final SWM contribution after integration over all quasi‐Bloch vector realizations as a dark blue cross. The speed of this movement is again constant and given by *V*τ, which is proportional to the spread of $\tilde{n}$ and therefore given by the tilt angle of the circle.

For the *t* ^2^‐contribution (violet) the situation is more involved because the Lissajous curve is less trivial. In this situation the crossing point of the two loops lies at v∼SWM,t2=(0,Im(p∼),0). Because of the vanishing occupation this point will not move in the following propagation and the Im$\left(\tilde{p}\right)$‐axis is again the symmetry axis of the dynamics. Consequently, the opposing Re$\left(\tilde{p}\right)$ values compensate each other, such that the final SWM contribution is again purely imaginary. Therefore, we have to consider the movement into the direction governed by the product n∼Re(p∼). When looking at the left column in Figure 10 the distribution reaches values with n∼Re(p∼)>0 (large dots) and n∼Re(p∼)<0 (small dots). Following Equation (13) the large dots move into the negative Im($\tilde{p}$)‐direction while the small ones move into the positive Im($\tilde{p}$)‐direction as indicated by the black horizontal arrows in the Im($\tilde{p}),\tilde{n}$‐plane at *t* < τ. In the complex $\tilde{p}$‐plane this leads to two counter movements marked as arrows in the right column. The small dots move up (dashed curved arrows) and the large dots move down (solid curved arrows). Initially both of these movements start with the same velocity because large and small dots have the same absolute values |n∼Re(p∼)|, that is, the Lissajous curve is symmetric. Therefore, the integrated SWM signal starts with a vanishing velocity for small *t* ≈ 0. However, during the following dynamics the quasi‐Bloch vectors with positive Im($\tilde{p}$)‐velocity (small dots) rotate collectively in such a way that this part of the curve obtains smaller occupations. This can be seen by focussing on the bottom‐left and upper‐right corners of the panels in the left column. Accordingly, those with negative Im($\tilde{p}$)‐velocity (large dots) obtain larger $|\tilde{n}|$ as can be seen in the second column [Im($\tilde{p}),\tilde{n}$‐plane] marked by the horizontal black arrows. In the left Lissajous curve this means that the large dots move toward the top left and bottom right corner of the plot, while the small dots move inwards. Consequently, the negative Im($\tilde{p}$)‐velocity component gains speed because |n∼Re(p∼)>0| (large dots) grows, while the positive component slows down as |n∼Re(p∼)<0| (small dots) shrinks. In the complex $\tilde{p}$‐plane (right column) this means that the upwards movement of the quasi‐Bloch vectors represented by small dots (dashed arrows) slows down for increasing times *t* while the downwards movement of the large dots (solid arrows) speeds up. In summary, for the integrated SWM coherence, marked by the violet cross, we find an acceleration towards negative Im($\tilde{p}$)‐values given by −*V* ^2^ *t* ^2^.

Exactly at *t* = τ in the third row the two integrated SWM contributions marked in the third and forth column have the same distance from the center as indicated by the dashed horizontal lines which show that they compensate each other. At this time the destructive echo appears due to the destructive quantum interference of all depicted quasi‐Bloch vectors. For even larger times *t* < τ (bottom row) the accelerated *t* ^2^‐contribution is stronger than the one with constant velocity ∼τ*t* and the SWM is non‐vanishing again.

## 3 Conclusion

To conclude we again compare the traditional photon echo in FWM with the newly discovered destructive photon echo in SWM. As summarized in **Table** **1** we define three criteria that characterize the echo formation. Firstly, the timing of the photon echo formation is basically determined by the chosen delay τ between the two laser pulses. Slight deviations from the exact *t* = τ timing are used to learn about internal dynamics of the studied system.^[^ ^51^ ^]^ The same holds for the destructive echo in SWM. As discussed in detail the two main contributions compensate each other exactly at *t* = τ. Nonetheless, in the detected and simulated full signal dynamics in Figure 4 the depression happens at *t* > τ. The reason is that exciton decay dynamically changes the slope of the destructive echo. Secondly, the photon echo appears because all considered Bloch vectors form a constructive interference. To explain this effect only the conventional FWM signal has to be considered, which would appear as a single path in a flow chart like the one in Figure 5 (see also ref. [43]). In the case of the destructive echo we have shown that we need two paths which interfere destructively to explain the novel echo effect. Finally, the fundamental source of the photon echo is any sort of inhomogeneity, which might appear in space by a locally varying exciton energy,^[^ ^52^ ^]^ or in time via external noise that acts on the optical transition energy.^[^ ^30^ ^]^ In the case of the destructive echo we have shown that the local field together with a variation of the applied laser pulse phases results in a spectral broadening. As discussed in ref. [43] this local field induced phase inhomogeneity can already be detected in FWM spectra. This summary shows that the criteria defining the traditional photon echo can also be applied to the destructive echo effect. Therefore, we conclude that we really found a new photon echo effect.

**Table 1:** Comparison of criteria defining the photon echo in FWM and the destructive photon echo in SWM

	Echo (FWM)	Destructive echo (SWM)
Timing	Delay + internal dynamics	Delay + internal dynamics
		
Interference	Constructive (1 path)	Destructive (2 paths)
		
Source	Inhomogeneous broadening	Local field induced phase inhomogeneity
		

In addition we have developed a quasi‐Bloch vector picture to illustrate the generation of the destructive echo inspired by the instructive Bloch vector image of the photon echo. We found that the SWM‐relevant quasi‐Bloch vectors are distributed along Lissajous curves which adds surprising aesthetics to the involved Bloch vector dynamics in SWM.

Following this proof‐of‐principle demonstration of a new photon echo effect the natural next tasks will be to further explore the destructive photon echo's application possibilities. One obvious step will be to measure photon echos at various spots of the sample and thereby get a measure for the spatial distribution of the local field coupling strength. We know that different pulse sequences in FWM probe different observables.^[^ ^53^ ^]^ Therefore we hope that novel pulse sequences in higher wave‐mixing spectroscopy will be designed that should allow to access parameters governing the nonlinear light‐induced dynamics in semiconductors, like the strength of interaction mechanisms, that are not easily accessible with traditional techniques. These developments are particularly attractive for materials with a large nonlinearity promoting strong wave mixing responses like TMDCs.

## 4 Experimental Section

In the experiment, we employ a mode‐locked laser (Tsunami Femto provided by *Spectra Physics*) generating femtosecond pulses at a repetition rate of 76 MHz. The center wavelength is tuned to 752 nm, which corresponds to the A exciton transition in the investigated hBN/MoSe_2_/hBN heterostructure at *T* = 5 K. The sample is kept in an optical He‐flow cryostat and microspectroscopy is performed using an Olympus microscope objective (*LCPLN50XIR*, numerical aperture of 0.65), which is installed on an XYZ piezo stage from Physik Instrumente. To perform the multi‐pulse, heterodyne experiments, the initial pulse train from the laser source is split into three and each of these beams is focussed into a separate acousto‐optic modulator (AOM). The AOMs are driven at distinct radio‐frequencies of Ω_1_ = 80, Ω_2_ = 79, and Ω_3_ = 79.77 MHz, such that the deflected beams acquire corresponding frequency upshifts. Next, the time delays between the first two (τ_12_) and the last two beams (τ_23_) are introduced by a pair of mechanical delay stages. In addition, a grating‐based pulse shaper is employed to correct the temporal chirp of the pulses, when passing through optical elements of the setup, especially AOMs and the microscope objective. The such prepared pulse sequence is then recombined into the same spatial mode and focussed at the heterostructure sample reaching a diffraction limited spot diameter of around 0.8 µm. The nonlinear optical signal from the sample is retrieved in the back‐reflectance geometry. To isolate the desired wave‐mixing response another AOM is used, operating at the heterodyne frequency generated by a home‐made analogue radio‐frequency mixing electronics, assembled from individual components provided by Mini‐Circuits. For example, the studied SWM signal is detected at (Ω_SWM_ = 2Ω_3_ − 2Ω_2_ + Ω_1_)=81.54 MHz. After being deflected from the AOM the signal is frequency downshifted by Ω_SWM_ and the unique wave‐mixing component under consideration carries the original frequency from the laser source. The signal can now interfere with a reference beam from the same laser source, which propagates in the vicinity of the driving beams. At the same time all other optical response components present in the signal reflected from the sample are still modulated in the MHz range and thus average out completely during a single acquisition time of a few ms. Importantly, the mixing AOM operates in a Bragg configuration. Hence, simultaneously the reference beam gets deflected onto the signal, receiving a frequency upshift by Ω_SWM_. This again allows for interference with the signal, which was not deflected by the AOM (operating at Ω_SWM_). In total, we generate two beams in which the considered wave‐mixing signal is interfering with the reference pulse, but with the opposite phase, as required by energy conservation. The spectrally‐resolved interference is obtained with an imaging spectrometer (Princeton Instruments with a focal length of 750 mm) and detected with an CCD camera (*PIXIS* from Princeton Instruments, with an eXcelon coating). A background free, shot‐noise limited detection is achieved by a balanced detection and multi‐acquisition. Namely, we exploit the Bragg configuration and subtract the two mixed beams impinging at different positions on the CCD. In addition, the phase of the mixing AOM is cycled between 0 and π to overcome any classical noise from the CCD camera. The wave‐mixing signal amplitude and phase are obtained from the interferogram by performing spectral interferometry.

## Conflict of Interest

The authors declare no conflict of interest.

## Supporting information

Supporting Information

## Data Availability

The data that support the findings of this study are available from the corresponding author upon reasonable request.
