# Supplementary material for: Destructive Photon Echo Formation in Six‐Wave Mixing Signals of a MoSe2 Monolayer
Source: Adv Sci (Weinh). 2021 Oct 29;9(1):2103813. doi: 10.1002/advs.202103813 (PMC8728888; doi:10.1002/advs.202103813)
Supplement: Supplementary file 1 — Supporting Information [file ADVS-9-2103813-s001.pdf]

ADVANCED SCIENCE
Open Access

Supporting Information

for *Adv. Sci.,* DOI: 10.1002/advs.202103813

Destructive photon echo formation in six-wave mixing signals of a $MoSe_2$ monolayer

*Thilo Hahn, Diana Vaclavkova, Miroslav Bartos, Karol Nogajewski, Marek Potemski, Kenji Watanabe, Takashi Taniguchi, Paweł Machnikowski, Tilmann Kuhn, Jacek Kasprzak, Daniel Wigger**

Destructive photon echo formation in six-wave mixing signals of a $MoSe_2$ monolayer (Supplementary Information)

Thilo Hahn,[1,2] Diana Vaclavkova,[3] Miroslav Bartos,[3,4] Karol Nogajewski,[5] Marek Potemski,[3,5] Kenji Watanabe,[6] Takashi Taniguchi,[7] Paweł Machnikowski,[2] Tilmann Kuhn,[1] Jacek Kasprzak,[8] Daniel Wigger[2,*]

[1] *Institut of Solid State Theory, University of Münster, 48149 Münster, Germany*
[2] *Department of Theoretical Physics, Wrocław University of Science and Technology, 50-370 Wrocław, Poland*
[3] *Laboratoire National des Champs Magnétiques Intenses, CNRS-UGA-UPS-INSA-EMFL, 38042 Grenoble, France*
[4] *Central European Institute of Technology, Brno University of Technology, 61200 Brno, Czech Republic*
[5] *Institute of Experimental Physics, Faculty of Physics, University of Warsaw, 02-093 Warszawa, Poland*
[6] *Research Center for Functional Materials, National Institute for Materials Science, Tsukuba 305-0044, Japan*
[7] *International Center for Materials Nanoarchitectonics, National Institute for Materials Science, Tsukuba 305-0044, Japan*
[8] *Université Grenoble Alpes, CNRS, Grenoble INP, Institut Néel, 38000 Grenoble, France*
[*] *daniel.wigger@pwr.edu.pl*

This document includes following content:

S1 All SWM contributions in the $\chi^{(5)}$-regime

S2 Impact of the local field strength

S3 Impact of excitation induced dephasing

S4 Impact of intervalley scattering

S5 Impact of a non-vanishing delay τ_{12}

S6 Impact of inhomogeneous broadening

S1 All SWM contributions in the $\chi^{(5)}$-regime

To find all phase combinations which lead to the SWM-phase, we go back to the transformation rules in Eqs. (2):

$$
\begin{aligned}
p^{+} &= p^{-}\cos^2\left(\frac{\theta}{2}\right) + \frac{i}{2}\sin(\theta)(1-2n^{-})e^{i\phi} \\
&\qquad + \sin^2\left(\frac{\theta}{2}\right)p^{-*}e^{i2\phi} \\
&\approx p^{-}\left(1-\frac{\theta^2}{4}\right) + i\frac{\theta}{2}(1-2n^{-})e^{i\phi} + \frac{\theta^2}{4}p^{-*}e^{i2\phi}\,,
\end{aligned}
\tag{S1a}
$$

$$
\begin{aligned}
n^{+} &= n^{-} + \sin^2\left(\frac{\theta}{2}\right)(1-2n^{-}) + \sin(\theta)\mathrm{Im}\left(p^{-}e^{-i\phi}\right) \\
&\approx n^{-} + \frac{\theta^2}{4}(1-2n^{-}) + \theta\mathrm{Im}\left(p^{-}e^{-i\phi}\right)\,,
\end{aligned}
\tag{S1b}
$$

where we also included second order contributions in the optical field, which will become relevant later. We again set the pulse areas of all three pulses to be equal $\theta_1 = \theta_2 = \theta_3 = \theta$. After the first pulse, up to $\mathcal{O}(\theta^2)$ we get

$$
p_1^{+} = i\frac{\theta}{2}e^{i\phi_1}\,, \tag{S2a}
$$

$$
n_1^{+} = \frac{\theta^2}{2}\,. \tag{S2b}
$$

After the second pulse the state reads up to $\mathcal{O}(\theta^3)$

$$
p_2^{+} = i\frac{\theta}{2}\left(e^{i\phi_1}+e^{i\phi_2}\right) - i\frac{\theta^3}{8}\left[e^{i\phi_1}+2e^{i\phi_2}+e^{i(2\phi_2-\phi_1)}\right]\,, \tag{S3a}
$$

$$
n_2^{+} = \frac{\theta^2}{2}[1+\cos(\phi_2-\phi_1)]\,. \tag{S3b}
$$

Anticipating the transformation of the third pulse, we see that from the third-order polarization ($\sim\theta^3$) only the part with the FWM-phase $2\phi_2-\phi_1$ needs to be taken into account. Thus, collecting all the linear polarizations and the FWM polarization we obtain with Eq. (5) the time evolution during the delay τ

$$
\begin{aligned}
p_3^{-} &= \left[i\frac{\theta}{2}\left(e^{i\phi_1}+e^{i\phi_2}\right) - i\frac{\theta^3}{8}e^{i(2\phi_2-\phi_1)}\right]e^{-\beta\tau} \\
&\qquad - iV\tau\left[i\frac{\theta}{2}\left(e^{i\phi_1}+e^{i\phi_2}\right)\right]\frac{\theta^2}{2}\cos(\phi_2-\phi_1)e^{-\beta\tau}\,, \\
&\to \left[i\frac{\theta}{2}\left(e^{i\phi_1}+e^{i\phi_2}\right) - i\frac{\theta^3}{8}e^{i(2\phi_2-\phi_1)}\right]e^{-\beta\tau} \\
&\qquad + V\tau\frac{\theta^3}{8}e^{i(2\phi_2-\phi_1)}e^{-\beta\tau}\,,
\end{aligned}
\tag{S4a}
$$

$$
n_3^{-} = \frac{\theta^2}{2}[1+\cos(\phi_2-\phi_1)]\,. \tag{S4b}
$$

Again, we have omitted all terms which will not contribute to the SWM signal in the $\chi^{(5)}$-regime. Note, that due to intervalley scattering, the occupation n_3^{-} is split between the valleys as discussed in the main text. The third pulse then transforms polarization and occupation into

$$
\begin{aligned}
p_3^{+} &= i\frac{\theta}{2}\left(e^{i\phi_1}e^{-\beta\tau}+e^{i\phi_2}e^{-\beta\tau}+e^{i\phi_3}\right) \\
&\quad - i\frac{\theta^3}{4}e^{i(\phi_3-\phi_2+\phi_1)} \\
&\quad - \frac{\theta^3}{8}e^{i(2\phi_2-\phi_1)}\left(i-V\tau\right)e^{-\beta\tau} \\
&\quad - i\frac{\theta^3}{8}\left[e^{i(2\phi_3-\phi_1)}+e^{i(2\phi_3-\phi_2)}\right]e^{-\beta\tau} \\
&\quad + \frac{\theta^5}{32}e^{i(2\phi_3-2\phi_2+\phi_1)}\left(i+V\tau\right)e^{-\beta\tau}\,,
\end{aligned}
\tag{S5a}
$$

$$
\begin{aligned}
n_3^{+} &= \frac{\theta^2}{2}\big[\cos(\phi_2-\phi_1)+\cos(\phi_3-\phi_1)e^{-\beta\tau} \\
&\qquad + \cos(\phi_3-\phi_2)e^{-\beta\tau}\big] \\
&\quad - \frac{\theta^4}{8}\cos(\phi_3-2\phi_2+\phi_1)e^{-\beta\tau} \\
&\quad - \frac{\theta^4}{8}V\tau\sin(\phi_3-2\phi_2+\phi_1)e^{-\beta\tau}\,.
\end{aligned}
\tag{S5b}
$$

Note, that we have again omitted all terms that are irrelevant for the final SWM signal, especially constant terms for the occupation that do not carry any phase information. Next, we consider the time-evolution after the third pulse, where we can already sort for the SWM phase $2\phi_3-2\phi_2+\phi_1$. In Fig. 5 in the main text we schematically depict the origin of the contributions with two local-field mixing processes for $\mathcal{O}(V^2)$. We disentangle the different contributions with $\mathcal{O}(V^1)$ and the SWM polarization from the pure TLS without local-field mixing with $\mathcal{O}(V^0)$ by the help of the flow charts in Fig. S1.

Starting with the polarization in (S5a) that already carries the SWM phase, their signal contribution reads

$$
{}^{\mathrm{SWM}}p/\theta^5 = \frac{i}{32}e^{-\beta\tau} + \frac{1}{32}V\tau e^{-\beta\tau}\,. \tag{S6a}
$$

The first part represents the SWM signal from the pure TLS as it is independent of V. Its origin is depicted in Fig. S1 on the left side (i) and we directly see that the coherence is detected when scanning the delay between the second and third pulse as mentioned in the main text. The second term (ii) is proportional to $V\tau$, therefore it stems from the local-field mixing between ${}^{\phi_2}p_2^{+}$ and ${}^{|\Delta_{21}|}n_2^{+}$ during the delay propagation, depicted is the most right path in Fig. S1.

From the FWM-polarizations created by the third pulse, we get the two SWM contributions (iii) and (iv) by a single local field mixing process in the real time propagation. According to Eq. (5) we have to calculate $p(t) = -iVtp_0n_0$ for single and $p(t) = -(Vt)^2p_0n_0^2/2$ for double mixing. (iii) is created by mixing the three-pulse FWM term ${}^{\Delta_{32}+\phi_1}p_3^{+} = {}^{\phi_3-\phi_2+\phi_1}p_3^{+}$

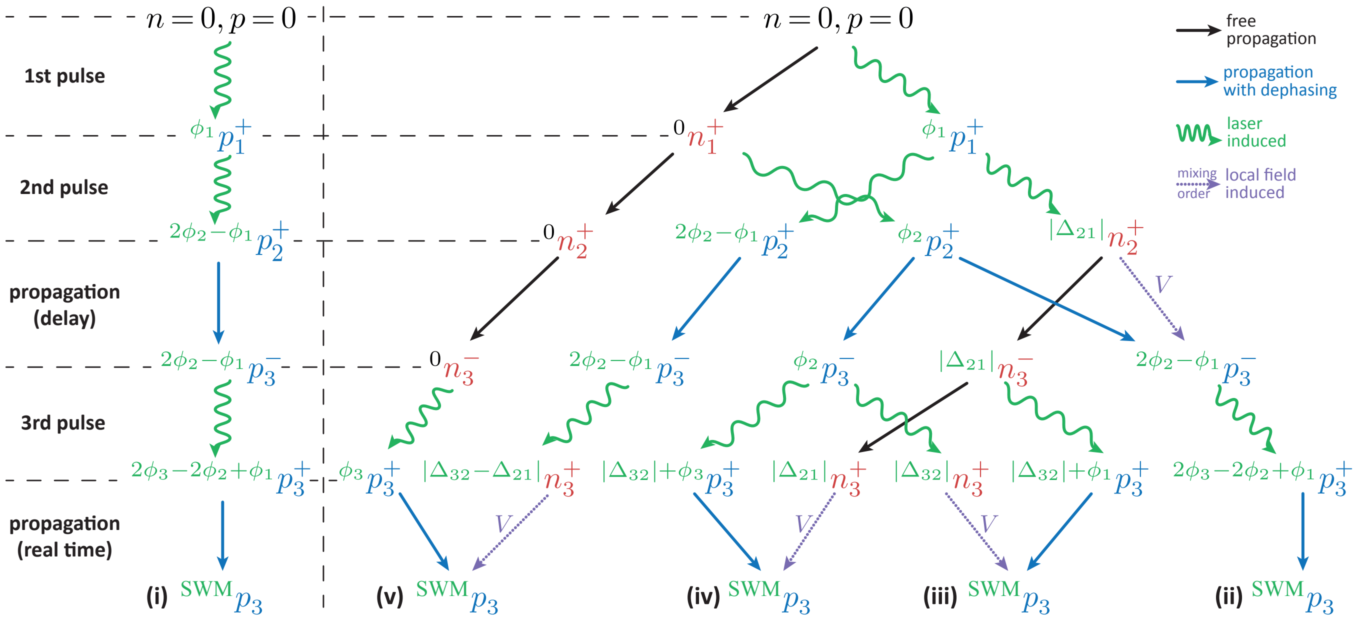

Figure S1: Flow charts for the SWM signal contributions without local field coupling (left) and proportional to V (right). Same as Fig. 5 in the main text. The labels (i) – (v) refer to the respective contributions in Eqs. (S6).

and ${}^{|\Delta_{32}|}n_3^+$ resulting in

$$\begin{aligned} {}^{\mathrm{SWM}}p/\theta^5 &= -iVt\left(-\frac{i}{4}\right)\left(\frac{1}{4}e^{-\beta\tau}\right) \\ &= -\frac{1}{16}Vte^{-\beta\tau}\,. \end{aligned} \tag{S6b}$$

The second one (iv) is created by the two-pulse FWM term ${}^{2\phi_3-\phi_2}p_3^+$ mixed with ${}^{|\Delta_{21}|}n_3^+$ leading to

$$\begin{aligned} {}^{\mathrm{SWM}}p/\theta^5 &= -iVt\left(-\frac{i}{8}e^{-\beta\tau}\right)\left(\frac{1}{4}\right) \\ &= -\frac{1}{32}Vte^{-\beta\tau}\,. \end{aligned} \tag{S6c}$$

Finally, contribution (v) is created by local field mixing ${}^{\phi_3}p_3^+$ with ${}^{|\Delta_{23}-\Delta_{12}|}n_3^+$ resulting in

$$\begin{aligned} {}^{\mathrm{SWM}}p/\theta^5 &= -iVt\left(\frac{i}{2}\right)\left(-\frac{1}{16}e^{-\beta\tau}\right) \\ &= -\frac{1}{32}Vte^{-\beta\tau}\,. \end{aligned} \tag{S6d}$$

For completeness we also give the three SWM contributions with $\mathcal{O}(V^2)$ here. As illustrated in Fig. 5 in the main text they are retrieved by double local field mixing between ${}^{\phi_1}p_3^+$ and ${}^{\Delta_{32}}n_3^+$

$$\begin{aligned} {}^{\mathrm{SWM}}p/\theta^5 &= -\frac{1}{2}(Vt)^2\left(\frac{i}{2}e^{-\beta\tau}\right)\left(\frac{1}{4}e^{-\beta\tau}\right)^2 \\ &= -\frac{i}{64}(Vt)^2e^{-3\beta\tau}\,, \end{aligned} \tag{S7a}$$

between ${}^{\phi_3}p_3^+$, ${}^{\Delta_{32}}n_3^+$, and ${}^{\Delta_{21}}n_3^+$

$$\begin{aligned} {}^{\mathrm{SWM}}p/\theta^5 &= -\frac{1}{2}(Vt)^2\left(\frac{i}{2}\right)2\left(\frac{1}{4}e^{-\beta\tau}\right)\left(\frac{1}{4}\right) \\ &= -\frac{i}{32}(Vt)^2e^{-\beta\tau}\,, \end{aligned} \tag{S7b}$$

where the factor 2 stems from the sum of the two occupations, and finally the single local field mixing between ${}^{\phi_3}p_3^+$ and ${}^{\Delta_{32}-\Delta_{21}}n_3^+$

$$\begin{aligned} {}^{\mathrm{SWM}}p/\theta^5 &= -iVt\left(\frac{i}{2}\right)\left(-\frac{1}{16i}V\tau e^{-\beta\tau}\right) \\ &= \frac{i}{32}V^2t\tau e^{-\beta\tau}\,. \end{aligned} \tag{S7c}$$

Collecting all the expressions finally yields

$$\begin{aligned} {}^{\mathrm{SWM}}p = \left(\frac{\theta}{2}\right)^5 \Big[& i + V(\tau-4t) \\ & -\frac{i}{2}(Vt)^2e^{-2\beta\tau} + iV^2t(\tau-t)\Big]e^{-\beta(t+\tau)} \end{aligned} \tag{S8}$$

for the SWM signal in the $\chi^{(5)}$-regime.

From Eq. (7)/(S8) we see that in the lowest order of the light-matter coupling, the signal amplitude scales with the fifth power of the pulse area. Therefore, operating in the low excitation regime the SWM dynamics do not depend on the choice of θ. To confirm this in Fig. S2 we plot the same SWM dynamics as in Fig. 4(b) in the main text but with halved (a) and doubled (b) pulse area. There are no obvious differences between the two simulations. This confirms that the chosen pulse area

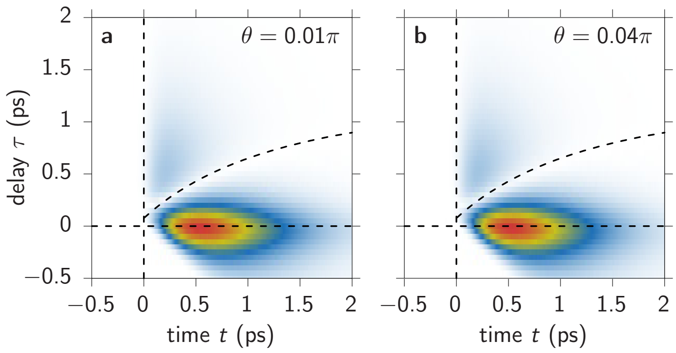

Figure S2: SWM dynamics for different pulse areas θ being halved (a) or doubled (b) compared to Fig. 4(b) in the main text.

does not affect the signal dynamics and that the determined local field coupling is a reasonable quantity to characterize the dynamics.

S2 Impact of the local field strength

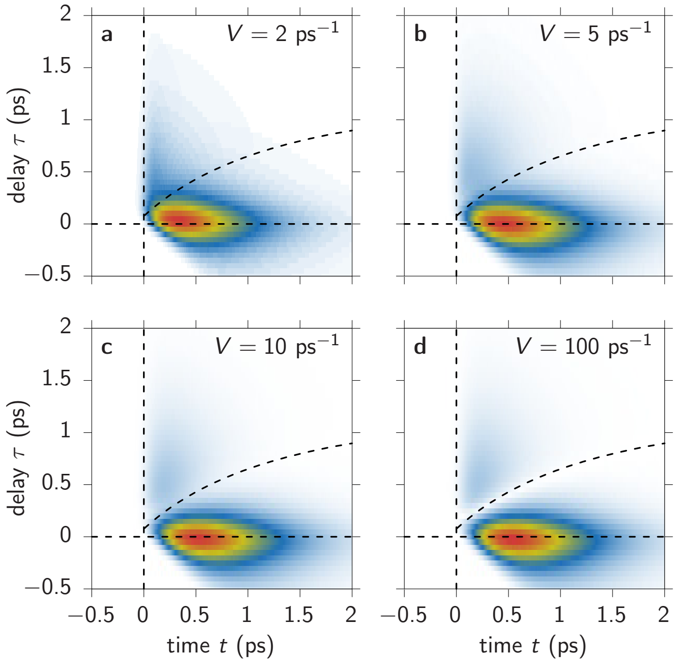

Figure S3: Comparison of the SWM dynamics for different local field strengths V increasing from (a) to (d), where (d) is the example from Fig. 4(b) in the main text.

From the analytical solution, we find the destructive echo only in the highest possible order, i.e., V^2. To understand, how the local field strength influences the destructive echo, in Fig. S3 we show the SWM dynamics for different values of V increasing from (a) to (d). While for $V = 2\,\text{ps}^{-1}$ (a) and $V = 5\,\text{ps}^{-1}$ (b) no destructive echo is visible, for $V = 10\,\text{ps}^{-1}$ (c), the depression is already visible. For comparison Fig. S3(d) shows the case with $V = 100\ \text{ps}^{-1}$ from the main text. This again confirms, that the V^2-order has to dominate the signal to observe the destructive echo. For $V = 10\,\text{ps}^{-1}$ in Fig. S3(c) we see that the destructive echo follows the dynamics of the minimum approximated by Eq. (9) shown as dashed curved line. This confirms our finding that this behavior is not affected by the strength of the local field coupling V.

S3 Impact of excitation induced dephasing

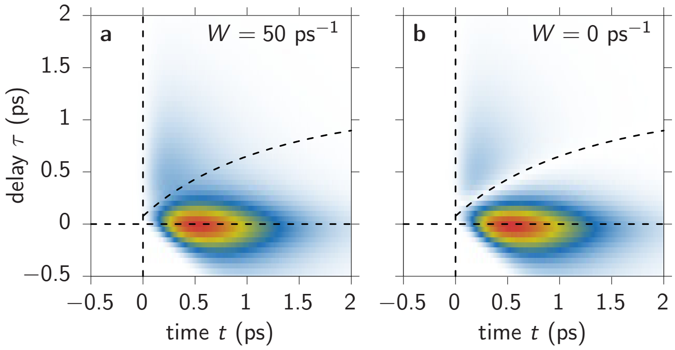

Figure S4: Impact of a non-vanishing excitation induced dephasing with $W = 50$ ps^{-1} (a) and (b) is the example from Fig. 4(b) in the main text with $W = 0$.

In addition to the local field coupling which shifts the transition energy depending on the exciton occupation, we can also take excitation induced dephasing (EID) into account [1], where the dephasing grows with the occupation $\beta_{\text{total}} = \beta + W(n + n')$. For an EID on the same order of magnitude as the local field the destructive echo effect is strongly suppressed as illustrated in Fig. S4(a) for $W = 50\,\text{ps}^{-1}$. As reference in Fig. S4(b) we show the case without EID ($W = 0$) from the main text [Fig. 4(b)]. Obviously, the EID prohibits the development of a destructive photon echo.

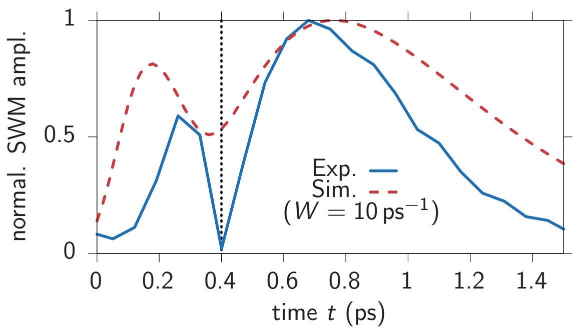

Figure S5: Impact of a non-vanishing excitation induced dephasing with $W = 10$ ps^{-1} on the SWM amplitude dynamics for $\tau = 0.35$ ps. Experiment as solid blue line and simulation as dashed red line. The measurement is the same as in Fig. 7(a).

In Fig. S5 we plot the same SWM amplitude dynamics as in Fig. 7(a) in the main text but in the simulation we

consider a EID of $W = 10$ ps^{-1}, which is much smaller than the applied local field coupling of $V = 100$ ps^{-1}. We see that the destructive photon echo minimum is significantly less pronounced when taking the EID into account. Therefore, to maintain the good agreement between theory and experiment, we have excluded EID from the discussion. We can conclude that the EID should not have a significant impact in our system.

S4 Impact of intervalley scattering

In the context of the approximation in Eq. (4b) we estimated that the intervalley scattering should only have an impact for small delays. To confirm this we calculate the SWM amplitude dynamics for $\lambda = 0$ as depicted in Fig. S6(a). Compared to Fig. 4(b) in the main text we cannot find significant deviations. To highlight the changes when setting $\lambda = 0$ in Fig. S6(b) we plot the difference with respect to the case with $\lambda = 4$ ps^{-1}. We indeed find, that the deviations are on the order of only $\approx 10\%$ and mainly restricted to delays $|\tau| < 0.5$ ps. Interestingly, we find that the amplitude difference has a change of sign that exactly follows the approximated evolution of the destructive echo minimum marked by the dashed line [see Eq. (9) in the main text]. Note, that the depicted color maps were calculated numerically employing the full model and taking a non-vanishing pulse duration into account.

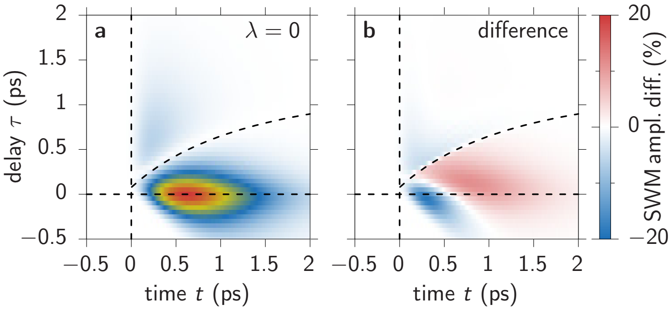

Figure S6: Impact of a vanishing intervalley scattering $\lambda = 0$ on the SWM amplitude dynamics. (a) SWM amplitude dynamics. (b) Difference of the SWM amplitude with respect to the case with $\lambda = 4$ ps^{-1} from Fig. 4(b) in the main text.

S5 Impact of a non-vanishing delay τ_{12}

In the main text we have considered a vanishing delay between the two pulses labeled with ϕ_1 and ϕ_2. As a reference the corresponding SWM dynamics are shown in Fig. S7(a) as functions of τ_{23}. To identify the impact of a non-vanishing delay between these two pulses, in Fig. S7(b) we plot the SWM dynamics for $\tau_{12} = 0.35$ ps. As depicted in the inset, for this delay the two pulses are almost entirely separated. The corresponding SWM amplitude dynamics are identical to the ones with $\tau_{12} = 0$ in Fig. S7(a) but have an overall smaller amplitude. This demonstrates that the destructive echo is not caused by pulse overlap effects. The reason for the decreased amplitude is, that the coherence ${}^{\phi_1}p_1^+$ after the first arriving pulse declines due to the considered dephasing. Consequently, the following processes, i.e., optical excitations and local-field mixing, are the same but start from a smaller coherence. Therefore, the final SWM signal is weaker.

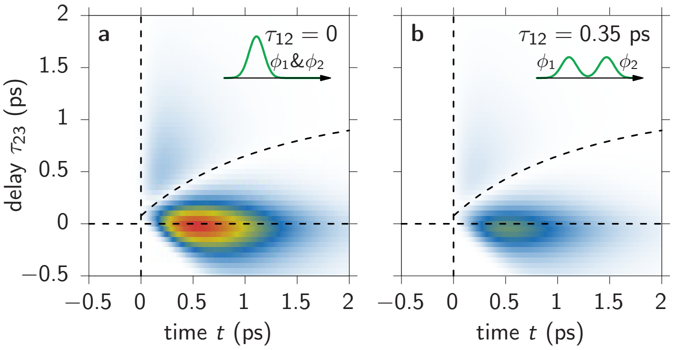

Figure S7: Impact of a non-vanishing pulse delay τ_{12} on the SWM amplitude dynamics. (a) Normalized SWM amplitude dynamics for $\tau_{12} = 0$ from Fig. 4(b) in the main text. (b) SWM amplitude dynamics for $\tau_{12} = 0.35$ ps normalized to the maximum value of (a). The insets schematically show the timing of the pulses with ϕ_1 and ϕ_2.

S6 Impact of inhomogeneous broadening

The experimental investigations of the sample do not show a significant inhomogeneous broadening, which should have manifested in the development of a photon echo in FWM and SWM. To demonstrate the impact of inhomogeneous broadening on the visibility of the destructive echo, we plot the destructive photon echo dynamics in Fig. S8(a), where the curved dashed black line indicates the minimum of the signal. In Fig. S8(b) we simulate the same SWM signal but set the local field coupling to $V = 0$ and consider an inhomogeneous broadening of $\sigma = 4$ ps^{-1} which leads to the development of a traditional constructive photon echo [2]. As marked by the two black dotted lines, the signal stretches along the diagonal $\tau = t$. Figure S8(c) combines both phenomena, showing the SWM including the original $V = 100$ ps^{-1} and the inhomogeneous broadening $\sigma = 4$ ps^{-1}. As indicated by the dashed and dotted lines, the destructive photon echo is still clearly visible but only in the time span of the traditional constructive photon echo.

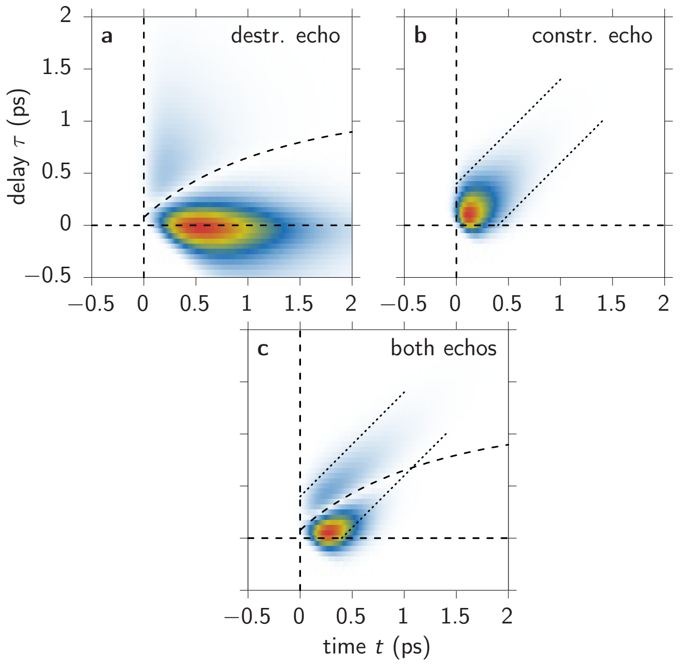

Figure S8: Impact of a non-vanishing inhomogeneous broadening on the SWM amplitude dynamics. (a) SWM amplitude dynamics with destructive echo from Fig. 4(b) in the main text. (b) SWM amplitude dynamics for $V = 0$ but with an inhomogeneous broadening of $\sigma = 4\ \mathrm{ps}^{-1}$ resulting in a constructive photon echo marked by the dotted black lines. (c) Combination of constructive and destructive photon echo. The signal depression is only visible in the vicinity of the constructive echo, while the entire signal is suppressed for all other times.

References

1. Rodek, A. *et al.* Local field effects in ultrafast light–matter interaction measured by pump-probe spectroscopy of monolayer $MoSe_2$. *Nanophotonics* **10**, 2717–2728 (2021).

2. Langbein, W. Coherent optical spectroscopy of semiconductor nanostructures. *Riv. del Nuovo Cim.* **33** (2010).
